# Effectiveness of a Smartphone App (Heia Meg) in Improving Decisions About Nutrition and Physical Activity: Prospective Longitudinal Study

**DOI:** 10.2196/48185

**Published:** 2024-04-30

**Authors:** Christine Olsen, Daniel Adrian Lungu

**Affiliations:** 1 Department of Quality and Health Technology Faculty of Health Sciences University of Stavanger Stavanger Norway

**Keywords:** app, BMI, diet, exercise, health, Heia Meg, lifestyle change, longitudinal, mHealth, mobile health, motivation, nutrition, obese, obesity, overweight, physical activity, smartphone apps, weight

## Abstract

**Background:**

Obesity is a prevalent and serious chronic condition associated with abnormal or excessive fat buildup that poses significant health risks. The rates of overweight and obesity in adults and children continue to rise, with global rates of children with overweight or obesity aged 5-19 years growing from 4% to 18% between 1975 and 2016. Furthermore, in 2017, nearly 4 million people died due to complications arising from being overweight or obese.

**Objective:**

This study aims to investigate the potential impact of the mobile app Heia Meg on promoting healthier lifestyle choices regarding nutrition and physical activity.

**Methods:**

A prospective longitudinal study was conducted in collaboration with the Norwegian Directorate of Health. Participants were recruited through the Heia Meg app and were asked to complete a questionnaire before and after using the app. A total of 199 responses were included in the first (preintervention) questionnaire, while 99 valid responses were obtained in the second (postintervention) questionnaire.

**Results:**

The majority (159/199, 79.9%) of participants were female, and their age ranged from 18 years to 70 years and older. The results show a reduction in BMI after the digital intervention. However, some variables influence the BMI reduction effect: sex, age, education, and smoking. The group that obtained the most benefit from the intervention consisted of those who were male, aged 30-39 years, highly educated, and nonsmokers. Although positive, some of the findings are slightly above the statistical significance threshold and therefore should be interpreted carefully.

**Conclusions:**

Our study found weak evidence to support the effectiveness of the Heia Meg app in promoting healthier lifestyle choices. However, limitations and confounding factors suggest that further research in different populations with larger sample sizes is needed to confirm or disprove our findings.

## Introduction

### Overview

Consumers have benefited from the internet’s transformation in information access about a decade ago [1]. Smartphones allow people to access information with a single touch. Smartphones offer a vast variety of health apps that users can download (for free or not). Many offer reference, monitoring, and calculator tools and address a wide range of health-related concerns. Apps count calories and nutrition, calculate BMI, monitor diabetes, handle emergencies, and improve workouts. Mobile health (mHealth) technology and consumer health informatics research have increased in recent years [1]. A survey by Sarcona et al [2] indicated that mHealth app users had significantly better eating behavior inventory scores or reported more positive eating behavior than nonusers. Fry and Neff [3] found that periodic reminders can aid in behavior improvement. These findings can be used to improve periodic quick interventions, leading to increased effectiveness, positive behavior change, and better health [3].

Health psychology models were used to design the experiment. Most social-cognitive theories presume that the desire to change predicts actual change, yet people rarely act on their intentions [4]. Heia Meg, which in Norwegian means the people’s conviction in their own capacities to produce predetermined levels of performance, influences the circumstances that affect their conduct. Belief in mastery influences people’s feelings, thoughts, motivation, and conduct. Cognitive, motivational, emotional, and selection processes generate this belief.

The health belief model (HBM) was devised in the 1950s to explain why so many people do not participate in disease prevention and detection programs [5]. The HBM is one of the most often used conceptual frameworks in health behavior research since 1950, “both to explain change and maintenance of health-related behaviors and as a guiding framework for health behavior treatments” [5,6]. If individuals regard themselves as susceptible to a condition, believing it could have serious consequences, while believing a course of action could reduce their susceptibility to or severity of the condition, and believing that the anticipated benefits of taking action outweigh the costs, this could increase the likelihood to act [5].

Perceived susceptibility refers to people’s health risks. Perceived severity refers to feelings about the seriousness of developing a disease or leaving it untreated, including medical and clinical implications (such as disability, death, and pain) and possible social consequences (such as effects on work, family life, and social relations) [5,7,8]. The individual’s thoughts regarding the perceived advantage of the potential activities for reducing the threat of illness can influence whether this perception leads to behavior change [5,7]. Other nonhealth factors, such as money savings from quitting smoking or pleasing a family member by obtaining a mammogram, may also influence behavior. Individuals with ideal views on susceptibility and severity are unlikely to choose a health intervention unless they believe it can lessen the threat. Perceived barriers are anticipated unfavorable outcomes of a health action. They may prevent people from engaging in the suggested activities. Individuals balance the action’s projected advantages against perceived barriers in an unconscious cost-benefit analysis. Consequently, “susceptibility and severity offer the energy or force to act, and benefits (without barriers) provide a preferred course of action” [5]. Cues to action are inputs that can trigger behaviors and were incorporated in previous HBM formulations. Hochbaum [9] argued that other aspects, such as physical occurrences or media attention, may only increase readiness to act (perceived susceptibility and perceived rewards).

Self-efficacy refers to having faith in one’s own strengths when presented with challenging activities and situations [10]. To attain mastery, one must learn from fresh hurdle situations and practice. A self-efficacy approach was implemented in the app messages as well. Through Heia Meg, messages are delivered as little challenges such as “Try something new, what about a walk before bedtime? Bring a headlamp, use the stairs, and take a 20-minute walk today” or sentences such as “You don’t like hills, yet they have something great to offer. Hills increase heart rate and health. So, imagine a top goal line and go for it.” These messages contain encouraging sentiments that may help people complete the challenge. The second most important source of self-efficacy is what we observe others do or accomplish. Positive role models can help develop positive self-beliefs and can include family, friends, teachers, coaches, or employers.

The third most important source of self-efficacy is social persuasion—receiving positive feedback while executing a difficult task might persuade a person, as they might start considering that they have the abilities and potential to succeed. The Heia Meg app’s daily messages have a positive tone, with statements such as “something is better than nothing.” or “The best session is the one you finish:)” or “A week has passed. Many people find it hard to start, so keep going. Cheers!” Since emotional and physiological factors might affect how someone feels about their ability, the app’s motivational sentences include “Good company helps when motivation fades.” “Know somebody who wants to get in shape? You can request a group activity.” “Before turning, take a 5-minute walk.” or “The first obstacle is often the hardest, but you can continue.”

**Figure 1:**
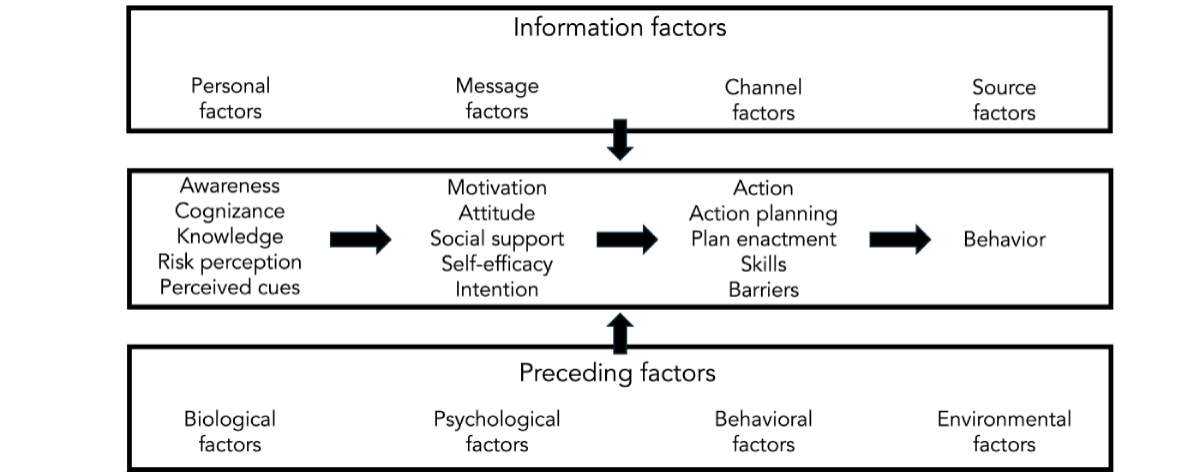
The I-Change model (2017).

The I-Change model was developed by Hein de Vries to explain health behavior and motivation [11]. I-Change combines aspects of the theory of planned behavior, the transtheoretical model for health behavior change, the social cognitive theory, the goal-setting theory, and the HBM to create a motivation and behavior change model. Motivation or intention determines behavior. Attitudes, social pressures, and self-efficacy affect motivation. Attitudes are the cognitive and emotional benefits and costs of an activity. Social modeling, social norms, and social support from others are examples of social influences on a person. New research suggests multiple types of self-efficacy, including stress-, social-, routine-, and skills-based self-efficacy. The I-Change model believes information and antecedent circumstances affect communication results—motivation, awareness, action, and behavior (Figure 1). Preparing and executing detailed plans to achieve the intended behavior increases the likelihood of intentions becoming actions, while barriers decrease these chances [11,12]. Attitudes, social pressures, and self-efficacy expectations all affect a person’s motivation.

### Overweight and Obesity

According to the World Health Organization, the number of 5- to 19-year-olds with overweight or obesity quadrupled between 1975 and 2016, and overweight and obesity cause more deaths than underweight [13]. We are also seeing a rise in overweight and obesity in Norway, largely due to an energy intake or consumption imbalance [14]. According to the Norwegian Directorate of Health, this increase is linked to increased inactivity, which can lead to various diseases over time, including increased mortality and increased risk of heart attack, stroke, high blood pressure, type 2 diabetes, several cancers, musculoskeletal disorders, and mental disorders. Another US study indicated that physical inactivity is the primary cause of increased mortality and morbidity in adults who are overweight (with a BMI up to 35 kg/m^2^), but that good physical shape can minimize the risk of weight-related diseases [15]. Increasing weight and obesity have health and economic effects, such as weight-related health problems accounting for 2% to 6% of overall health costs [16].

### mHealth

mHealth is a new sector with the potential to reach a large portion of the population cost-effectively. It is a branch of eHealth that uses technology to improve people’s health. Research shows that 2.9% of Google Play apps and 8.8% of Apple App Store apps support healthy behaviors [17]. Cost-effective technologies that record a user’s behavior are gaining prominence. Despite apps’ potential to alter health behavior, there is no proof of their health theory foundation [17]. According to Antezana et al [17], health apps have low levels of theory of behavior change strategies, and improved implementation could lead to more user engagement and better interventions. Mateo et al [18] compared a mobile app to other weight-loss and exercise strategies. In their review of 12 studies, using a mobile app positively affected body weight, but not physical activity.

### Heia Meg

The Heia Meg app was developed by the Norwegian Directorate of Health with inspiration from the United Kingdom’s “One You” and a previous Norwegian app called “Slutta” or “Quit” [19]. The project is nondirectly supportive and based on the idea that motivation changes behavior. The “Quit” app is a popular tool for Norwegians trying to quit smoking. Due to the “Quit” app’s success, self-efficacy and positive psychology were also used in Heia Meg.

Heia Meg self-efficacy examples include inspiring health alternatives, push-notification support, and push warnings preventing temptation.

The pursuit of happiness is one of the humanities’ most persistent movements [20]. Positive psychology aims to answer the question “What is happiness?” The Heia Meg app uses positive psychology by focusing on stress management and by pushing alerts encouraging users.

Push alerts are regular and scheduled. After downloading the app, users must agree to its terms before choosing 2 of 5 themes to focus on. Users can choose between (1) exercise, (2) mental health, (3) alcohol, (4) sleep habits, and (5) dietary intake. After choosing the 2 themes, the app will send notifications on a regular basis. The notifications include encouragements, theme facts, challenges, and recommendations. The texts are short and written in Norwegian, with no emojis or abbreviations.

The National Institute of Public Health (NIPH) national public health survey presented a report of results on diet, self-reported weight, and weight development in the Norwegian population in 2020 [21]. The report showed that over two-thirds of people wished to reduce weight or had tried to maintain their weight, as well as a large proportion of the population being overweight or obese. According to the findings of the public health survey on body weight and development, male individuals’ average weight, height, and BMI were 86.6 kg, 180.7 cm, and 26.5 kg/m^2^, respectively [21]. The percentage of male individuals who were overweight or obese (BMI ≥25 kg/m^2^) was 59%, while the percentage of male individuals who were underweight (BMI <18.5 kg/m^2^) was 0.8%. On the other hand, the average weight, height, and BMI for female individuals were 71.6 kg, 167.3 cm, and 25.6 kg/m^2^, respectively. The number of female individuals who were overweight or obese was 47%, while the proportion of female individuals who were underweight was 2.7%. Overall, 16% of female and male individuals had a BMI of 30 kg/m^2^ or higher, which indicates obesity [21]. The average person consumed 2 servings of fruits and vegetables each day, including juice, and according to the survey, only 2.3% reported that they consume at least 5 servings each day. Furthermore, the report stated that 43% of the participants ate fish 2-3 times per week, whereas 7% rarely or never ate fish. Approximately 30% said they ate sweets frequently (at least 3 times per week), while 10% reported eating snacks and 11% reported eating sweet pastries at least 3 times per week [21]. A large percentage of people reported using soft fat or a combination of soft and hard fat types or none for bread, and 61% reported using soft fat, oil, or nonfat for frying. Further, when looking at sugary soft drinks, juices, and soda, 13% reported drinking them 3 times a week or more, with the proportion being highest among younger male individuals and among the least educated. Dietary changes were also mentioned, with 5.4% following a low-carb diet, 3.8% following a calorie-reduced diet, and 3.3% fasting on a regular basis [21].

The survey revealed that Norway’s population needs and wants to improve its lifestyle regarding nutrition and weight loss. Therefore, the aim of this study was to test whether the Heia Meg app can help achieve this goal. Therefore, the following research question was formulated: “Is Heia Meg, a smartphone app, effective in helping users to make healthier lifestyle decisions about nutrition and physical activity?”

## Methods

### Overview

Based on past observations using the app and its structure and functioning, we anticipated that it could improve the physical activity behavior and eating habits of users. To test the assumption, we used a prospective longitudinal design. All statistical analyses were conducted using SPSS Statistics (version 26; IBM Corp).

### Recruitment Procedure

In collaboration with the Norwegian Directorate of Health, we added a link to the questionnaire within the Heia Meg app. This was only accessible to Heia Meg app users with internet connectivity enabled. Besides the in-app advertisement, posters were put on Facebook, Instagram, and Snapchat to foster recruitment. A poster was affixed to the wall of a neighboring fitness center, which contained complete information about the study and how to participate. Because the app and survey were written in Norwegian, so were the posters.

### Participants

The Heia Meg app was the only channel for the registration of study participants. The sample consisted of users who downloaded the app and agreed to take part in the study.

Everyone who downloaded the app between October 4, 2021, and November 5, 2021, received a message the day after downloading it asking whether they were interested in joining a research study. Participants had to read and sign a consent form confirming that they were 18 years of age or older and that they consented to participate. Participants were asked to provide their email address because that is what was used to send the follow-up survey.

### Materials

This project’s questionnaire was based on Harris’s [22] NIPH study, “Social circumstances and health: a twin study.” The form was modified for this investigation, and it contains 12 health-related items.

The 12 items response alternative were all defined as a Likert scale, so they could be graded from 1 to 4, with 1 being the worst and 4 being the best. The replies to the 12 items would be used to compute a health score. The score is the sum of the participants’ responses, with a minimum of 12 points (scoring 1 on all 12 questions) and a maximum of 48 points (scoring 4 on all 12 questions). This study took place during the COVID-19 pandemic, which may have affected people’s health, lifestyle, eating, drinking, and mental health.

### Ethical Considerations

An application, containing the project plan and the questionnaire, was submitted to the Norwegian Center for Research Data (NSD) and the regional ethics committee. As no health or sensitive data were going to be processed, the regional ethics committee confirmed that the project is approved without a formal assessment (application 284804).

Participants had to read and sign a consent form confirming that they were 18 years of age or older and that they consented to participate.

Most of the ethical considerations concern the gathering of personal data and how to organize, implement, and complete it in line with legislative requirements and ethical norms. The Health Research Act, the Health Register Act, laws on population-based health assessments, and the Privacy Ordinance and Personal Data Act offer a comprehensive foundation for medical and health research [23]. For this consent-based research project, the duty to disclose information is accomplished through Nettskjema. All personal information was to be anonymized for data processing; hence, a participant identification number was required. Due to participants using their email, their answers were not anonymous; nonetheless, the data were secured, anonymized, and held until the manuscript was submitted, following which they were deleted.

NSD approved the study in relation to the processing of personal data (approval 385157).

No compensation was given to the participants; they volunteered to take part in the study.

## Results

### Overview

We received 365 responses, but some did not meet the criteria and were eliminated. In the preintervention questionnaire, 5 people were removed for being 17 years of age or younger, 9 for refusing informed consent, and 20 for answering the questionnaire twice or more. Two people, aged younger than 18 years and 31 years, respectively, had repeated responses, which were eliminated from the postintervention questionnaire. This study included 298 replies in total: 66.78% (n=199) in the preintervention group and 33.22% (n=99) in the postintervention group.

### Descriptive Statistics

As depicted in Table 1, female individuals participated more than male individuals. Participants ranged in age from 18 years to 70 years and older, with the majority between 40-49 years (54/199, 27.1%) and 18-29 years of age (43.199, 21.6%). Most participants (63.199, 31.7%) had 4 or more years of college or university. Many (54/199, 27.1%) possessed high school diplomas or apprenticeship certificates. Male individuals have a higher BMI; the mean BMI for female individuals (29.33 kg/m^2^) falls under the overweight category, while male individuals (31.22 kg/m^2^) fall under the grade I obesity category. Furthermore, when looking at health score by sex, education, smoking, lifestyle change reason, and BMI, male individuals had a higher health score than female, while female individuals had a higher SD. The group of 30- to 39-year-olds had the highest health score, followed by those over 70 years old. The group of 60- to 69-year-olds had the lowest health score. Higher-educated people got better health scores than individuals with less education. Smokers had a poorer health score than nonsmokers. Comparing BMI and health score, those with the lowest BMI had the highest health score.

Table 2 displays the characteristics of respondents to the postintervention questionnaire. Again, female individuals were more represented than male individuals (76/99, 76% vs 23/99, 23%), and the age groups of 40- to 49-year-olds (22/99, 22%) and 18- to 29-year-olds (21/99, 21%) had the most respondents. We observed that some participants belonging to the 70 years or older age group were still using the app and continued to take part in the study. Male and female individuals’ mean BMI after 1 month with the app show that male individuals have a marginally higher BMI than female, while female individuals have a higher SD. People who were 70 years of age or older had the highest health score; however, there were only 5 participants in this group, and therefore precaution is needed in interpreting this result. Interestingly, the participant group of 18- to 29-year-olds had the lowest health score. People with higher education had a better health score than those with lower education. Nonsmokers had better health score than smokers.

The health score scale’s Cronbach α was .92, indicating good reliability.

For the following phase, we focus on pre-post analyses. We matched respondents from both the pre- and postintervention surveys, leaving us with a sample of 74 participants.

A paired sample 2-tailed *t* test examined BMI and health score pre-post differences. Results are presented in Table 3 below.

The data indicate that the BMI and the health score before intervention were higher than after intervention. More specifically, the differences between the pre- and postintervention groups were 0.284 for the BMI and 0.797 for the health score, as displayed in Table 4 below.

However, the results of the paired sample *t* test of the BMI and health score before and after intervention were not statistically significant (*P*=.07 for the BMI and *P*=.24 for the health score).

**Table 1:** Preintervention descriptive statistics of the participants recruited through Heia Meg: sex, age group, education, BMI, smoking status, reason for lifestyle change.

Characteristics			Participants (n=199), n (%)		Value, mean (SD)
**Sex**					
	Male		40 (20.1)		N/A^a^
	Female		159 (79.9)		N/A
	Prefer not to answer		0 (0)		N/A
**Age group (years)**					
	18-29		43 (21.6)		N/A
	30-39		32 (16.1)		N/A
	40-49		54 (27.1)		N/A
	50-59		36 (18.1)		N/A
	60-69		26 (13.1)		N/A
	70 or older		8 (4)		N/A
**Education**					
	Primary school		32 (16.1)		N/A
	High school or certificate of apprenticeship		54 (27.1)		N/A
	University (less than 4 years)		50 (25.1)		N/A
	University (more than 4 years)		63 (31.7)		N/A
**BMI (kg/m^2^) by**					
	**Sex**				
		Male	40 (20.1)	31.22 (6.34)	
		Female	159 (79.9)	29.33 (5.72)	
		Prefer not to answer	0 (0)	0 (0)	
		Total	199 (100)	29.71 (5.91)	
	**BMI groups (kg/m^2^)**				
		<18.5 (underweight)	0 (0)	0 (0)	
		18.5-24.9 (normal weight)	66 (33.2)	22.75 (1.60)	
		25.0-29.9 (overweight)	61 (30.7)	27.57 (1.53)	
		30.0-34.9 (obesity grade I)	48 (24.1)	32.24 (1.28)	
		35.0-39.9 (obesity grade II)	12 (6)	36.45 (2.53)	
		≥40 (obesity grade III)	12 (6)	43.34 (3.35	
**Health score by**					
	**Sex**				
		Male	40 (20.1)	36.90 (6.56)	
		Female	159 (79.9)	33.11 (7.69)	
		Prefer not to answer	0 (0)	0 (0)	
		Total	199 (100)	33.87 (7.62)	
	**Age group (years)**				
		18-29	43 (21.6)	33.77 (8.61)	
		30-39	32 (16.1)	36.25 (7.60)	
		40-49	54 (27.1)	32.96 (7.65)	
		50-59	36 (18.1)	33.56 (6.71)	
		60-69	26 (13.1)	32.77 (6.40	
		70 or older	8 (4)	36.00 (9.07	
		Total	199 (100)	33.87 (7.62)	
	**Education**				
		Primary school	32 (16.1)	31.25 (6.05)	
		High school or certificate of apprenticeship	54 (27.1)	32.07 (7.67)	
		University (less than 4 years)	50 (25.1)	34.08 (7.10)	
		University (more than 4 years)	63 (31.7)	36.57 (7.93)	
		Total	199 (100)	33.87 (7.62)	
	**Smoking**				
		Yes	18 (9)	31.56 (4.93)	
		No	181 (91)	34.10 (7.80)	
		Total	199 (100)	33.87 (7.62)	
	**Reason for lifestyle change**				
		Better sleeping habits	44 (12)	33.55 (8.37)	
		Drink less alcohol	17 (4.6)	35.53 (7.33)	
		Get in better shape	110 (30.1)	32.80 (7.85)	
		Make better nutrition choices	114 (31.1)	33.72 (7.09)	
		Mental health	81 (22.2)	34.22 (7.64)	
	**BMI (kg/m^2^)**				
		<18.5 (underweight)	0 (0)	0 (0)	
		18.5-24.9 (normal weight)	66 (33.2)	38.18 (7.60)	
		25.0029.9 (overweight)	61 (30.7)	34.30 (7.02)	
		30.00-4.9 (obesity grade I)	48 (24.1)	30.67 (5.04)	
		35.0-39.9 (obesity grade II)	12 (6)	26.67 (4.92)	
		≥40 (obesity grade III)	12 (6)	28.00 (7.03)	

^a^N/A: not applicable.

**Table 2:** Postintervention descriptive statistics of the participants recruited through Heia Meg: sex, age group, education, BMI, smoking status, reason for lifestyle change.

Characteristics			Participants (n=99), n (%)		Value, mean (SD)
**Sex**					
	Male		23 (23)		N/A^a^
	Female		76 (77)		N/A
	Prefer not to answer		0 (0)		N/A
**Age group (years)**					
	18-29		21 (21)		N/A
	30-39		17 (17)		N/A
	40-49		22 (22)		N/A
	50-59		17 (17)		N/A
	60-69		17 (17)		N/A
	70 or older		5 (5)		N/A
**Education**					
	Primary school		8 (8)		N/A
	High school or certificate of apprenticeship		28 (28)		N/A
	University (less than 4 years)		24 (24)		N/A
	University (more than 4 years)		39 (39)		N/A
**BMI (kg/m^2^) by**					
	**Sex**				
		Male	23 (23)	28.62 (5.25)	
		Female	76 (77)	28.52 (5.98)	
		Prefer not to answer	0 (0)	0 (0)	
		Total	99 (100)	28.54 (5.79)	
	**BMI groups (kg/m^2^)**				
		<18.5 (underweight)	0 (0)	23.24 (1.45)	
		18.5-24.9 (normal weight)	35 (35)	27.22 (1.68)	
		25.0-29.9 (overweight)	29 (29)	32.19 (1.28)	
		30.0-34.9 (obesity grade I)	23 (23)	36.7 (1.38)	
		35.0-39.9 (obesity grade II)	5 (5)	42.7 (1.67)	
		≥40 (obesity grade III)	7 (7)	44.34 (1.55)	
**Health score by**					
	**Sex**				
		Male	23 (23)	35.09 (7.37)	
		Female	76 (77)	32.25 (7.90)	
		Prefer not to answer	0 (0)	0 (0)	
		Total	99 (100)	32.91 (7.84)	
	**Age group (years)**				
		18-29	21 (21)	30.71 (8.69)	
		30-39	17 (17)	32.65 (7.57)	
		40-49	22 (22)	33.41 (8.99)	
		50-59	17 (17)	33.35 (7.57)	
		60-69	17 (17)	32.29 (4.81)	
		70 or older	5 (5)	41.40 (5.77)	
		Total	99 (100)	32.91 (7.84)	
	**Education**				
		Primary school	8 (8)	32.25 (6.56)	
		High school or certificate of apprenticeship	28 (28)	31.07 (8.13)	
		University (less than 4 years)	24 (24)	32.00 (7.00)	
		University (more than 4 years)	39 (39)	34.92 (8.16)	
		Total	99 (100)	32.91 (7.84)	
	**Smoking**				
		Yes	8 (8)	26.25 (8.48)	
		No	91 (92)	33.49 (7.55)	
		Total	99 (100)	32.91 (7.84)	
	**Reason for lifestyle change**				
		Better sleeping habits	25 (14)	32.52 (9.47)	
		Drink less alcohol	10 (6)	33.60 (6.90)	
		Get in better shape	54 (31)	31.44 (7.86)	
		Make better nutrition choices	47 (27)	34.02 (7.09)	
		Mental health	40 (23)	32.63 (8.38)	
	**BMI (kg/m^2^)**				
		<18.5 (underweight)	0 (0)	0 (0)	
		18.5-24.9 (normal weight)	35 (35)	35.91 (7.15)	
		25.0029.9 (overweight)	29 (29)	31.24 (7.06)	
		30.00-4.9 (obesity grade I)	23 (23)	32.87 (7.96)	
		35.0-39.9 (obesity grade II)	5 (5)	37.20 (5.02)	
		≥40 (obesity grade III)	7 (7)	21.86 (2.85)	

^a^N/A: not applicable.

**Table 3:** Paired sample 2-tailed t test statistics of BMI and health score.

		Value, mean (SD)	Value, SE mean
**BMI (n=74)**			
	Preintervention	29.05 (5.45)	0.634
	Postintervention	28.77 (5.38)	0.626
**Health score (n=74)**			
	Preintervention	34.65 (7.11)	0.826
	Postintervention	33.85 (6.96)	0.809

**Table 4:** Paired sample 2-tailed t test and paired differences of BMI and health score.

	Value, mean (SD)	Value, SE mean	Lower-upper, 95% CIs	*t* test (*df*)	*P* value
BMI	0.284 (1.32)	0.154	–0.023 to 0.590	1.84 (73)	.07
Health score	0.797 (5.82)	0.676	–0.551 to 2.145	1.179 (73)	.24

## Discussion

### Participants

The majority of study participants were female, 79.9% (159/199) before intervention and 77% (76/99) after intervention. Is it because female individuals are more interested in improving their health or have more faith in health-related apps or the app’s design? This is a question that deserves further investigation. Another intriguing observation was the high percentage of participants aged 40-69 years (116/199, 58.3% for the preintervention questionnaire and 56/99, 57% for the postintervention questionnaire), as well as the participation of people aged 70 years and older (8/199, 4% and 5/99, 5%, respectively). When the questionnaire was delivered through Heia Meg, it was assumed that younger people would use it. Instead, we believe that for older individuals, the fact that the app was produced and sponsored by the Norwegian Directorate of Health was reassuring and trustworthy. The relatively small sample size in our study does not allow us to generalize these findings.

Most of the study participants had a higher education (113/199, 56.8% in the preintervention group and 63/99, 64% in the postintervention group). The link between the degree of education and health literacy and the engagement of individuals in their health, including participation in research studies such as ours, is worth discussing further. Furnée et al [24] conducted a meta-analysis to determine the marginal impact of education on self-reported health, while Nummela et al [25] examined the relationship between self-reported health and 3 variables of social economic position (disposable household income, self-reported education, and adequacy of income) and 3 categories of communities (rural areas, highly or sparsely papillated areas, and urban areas) among male and female individuals in southern Finland. The adequacy of income had the strongest positive connection with self-reported health in metropolitan locations among all age groups, demonstrating that while real income is a powerful predictor of health, the adequacy of income is even greater [25]. Furnée et al [24] found that a year of education added 0.036 quality-adjusted life-years. Preliminary calculations show that investing in education improves health.

Individuals with high health literacy may make better decisions regarding their health and well-being [26]. Environmental needs and available resources affect health literacy, highlighting the need to enhance it in schools and communities [27]. Individually, health literacy efforts in schools should focus on teaching meta-cognitive abilities such as critical thinking, self-awareness, and citizenship. Health literacy education should include socioeconomic determinants of health and societal processes that lead to health inequities [27].

Most of our study participants wished to improve their diet and fitness. Male individuals’ mean BMI was higher than female individual’s (31.2 kg/m^2^ vs 28.6 kg/m^2^ preintervention and 29.3 kg/m^2^ vs 28.5 kg/m^2^ postintervention). Respondents in the preintervention group had mostly type I obesity, while female individuals were overweight. The intervention proved beneficial in lowering the mean BMI of study participants. Male individuals’ mean BMI decreased more than female individual’s, and in the postintervention survey, both sexes were overweight. After intervention, both sexes ended up in the overweight category as a mean BMI; several participants were within the BMI category of normal weight; none of the participants were underweight; 10 participants were overweight; and 7 had obesity (group III).

Male individuals had a higher health score than female individuals, both in the preintervention observation (36.9 vs 35.09) and in the postintervention questionnaire (33.1 vs 32.25). Interestingly, both sexes experienced a reduction in their health scores between the pre- and postintervention questionnaires. One possible interpretation of this result is that participants gained self-awareness between the 2 questionnaires, and their replies in the postintervention phase were more realistic than preintervention. Moreover, this study was conducted during the COVID-19 pandemic, and society was locked down and reopened numerous times. We foresee the possibility that the fact that Norwegian society was more open when the study began (October 4, 2021) than when the postintervention questionnaire was sent out (November 25, 2021) could have impacted our results. We all experienced changes in our behavior during the lockdown times of the COVID-19 pandemic, and the same could be true for the participants in this study. In a study conducted during the COVID-19 pandemic, Flanagan et al [28] found that eating behavior altered significantly. Cooking at home rose from 4.49 to 5.18 times per week (*P*<.001), while eating out fell from 1.98 to 1.08 (*P*<.001). Fast-eating assessments improved from 0.04 to 0.81 (*P*<.001), indicating a healthier diet. A total of 35.6% of the participants ate unhealthier food in general, and 43.5% ate more unhealthy snacks [28]. Those who ate poorly had a more sedentary lifestyle, less physical exercise, did not go to bed or sleep later, and reported almost doubling in anxiety [28].

When looking at the health score stratified by age, the group of 30- to 39-year-olds had the highest health score before intervention but one of the lowest after interventions. For those 70 years of age and older, their preintervention health score was 36 and 41.40 after intervention. This age group has more stability in their lives, which influences their perceived health, diet, and exercise habits. However, we must highlight that this age group accounted for less than 5% (13/298) of the study sample; therefore, the findings are not generalizable, and further research is needed to confirm these findings or not.

As anticipated within our research group based on existing evidence, we found that those with the lowest level of education also had the lowest health score, while those with the highest level of education had the highest health score. Smokers have poorer health scores than nonsmokers, and this finding is not unexpected considering available evidence. Tobacco use has long been linked to life-threatening diseases [29], and it is one of the top 10 practices that cause global sickness [5,29].

In most low-income nations, socioeconomic status creates health disparities between persons of different income levels. Chronic diseases and behavioral risk factors are more widespread in low-income and low-educated people [30]. Tobacco, sedentary habits, poor diets, and alcohol cause 1 million deaths per year in the United States alone, and changing health behaviors is our best hope for reducing global disease and death [5]. Socioeconomic causes and reducing health disparities should be prioritized by public health officers and policy makers [30].

We presented in the *Results* section the difference in BMI and health score before and after using the app. We saw that the mean preintervention BMI was higher than the mean postintervention BMI, even though the difference was not statistically significant (*P*=.07). Given the positive change observed in the BMI, while being aware that there are multiple factors beyond the app that could have influenced this change (eg, motivation), we encourage further research extended to larger samples to investigate the effectiveness of similar apps on the BMI.

This project’s goal was to contribute to digital health app research. Over 200 people joined the study through the app, and a response rate of 49.7% (99/199) was observed in the postintervention questionnaire. Participants were given the possibility of making suggestions about the design and functionality of the app. Some of their feedback messages are similar to the following: “Should have been more specific tips”; “I only get push alerts from Heia Meg, not alerts with a visible note, that’s what I need to get motivated from the app”; “Make it clear, what extreme progression you get when first starting a lifestyle change as physical activity, as well as how these progression values for everyday life”; “It’s motivating to motivate others, and some counsel is too simplistic”; or “It’s great, simple, and uncomplicated.” We used the feedback and reported it to the Norwegian Directorate of Health, which used it as a quality improvement tool.

Although the positive effects we observed were not statistically significant, we cannot exclude that this was due to an insufficient statistical power obtained from our relatively small sample and therefore encourage more research from other scholars who have access to other pools of participants.

When a program or intervention is motivated by a health behavior theory, individuals and communities benefit [5]. Never before have health education and behavior change workers had so many possibilities. Mobile phones, laptops, and smartphone apps have the potential to help people improve their lifestyles. Digital interventions can improve awareness of dangers or benefits, provide cues or reminders for healthy habits, and provide encouragement or training to boost confidence. Digital interventions can help increase self-efficacy through mastery and vicarious experiences, as well as social persuasion, emotional, and physiological factors with sending prompts and challenges for engaging in healthy behaviors, as well as the opportunity to follow friends, family, or a role model.

### Limitations

When recruiting participants, the survey’s inadequacies harmed the data’s validity and dependability. Several participants modified their behavior aim between the first and second questionnaires, reducing the number of participants for a 2-tailed paired *t* test. In other cases, people filled out the questionnaire multiple times, giving different ages, education levels, and themes. The questionnaire was not sent out until the day after the app was downloaded, but the recruitment of participants pulled in individuals who were truly using the app, as they had to read the message and click the link to the questionnaire. Our recruitment strategy yielded more female than male individuals, while a more balanced sample could have been more generalizable to the general public. Our sample seems skewed toward higher education, and as for sex, a more balanced sample would make it easier to extend our finding to the general public.

The study’s shortcomings include a lack of a control group to determine the cause and effect of the Heia Meg app, as several confounding factors may have influenced our results (eg, the motivation of each participant and all the exogenous factors). Voluntary participation and a restricted number of participants may risk the study’s external validity, as volunteers may have different opinions than the general public.

### Conclusions

This research project attempted to investigate the effectiveness of the lifestyle app Heia Meg, developed by the Norwegian Directorate of Health, in helping individuals make healthier lifestyle decisions about nutrition and physical activity. Although we did observe some positive effects, the differences were not statistically significant, and therefore we conclude that from our study, it does not appear that the app intervention leads to better nutrition and physical activity choices. This result could be affected by the limitations and confounding factors described above, and further research is needed to confirm, or not confirm, this conclusion.

## Abbreviations

HBM: health belief model
mHealth: mobile health
NIPH: National Institute of Public Health
NSD: Norwegian Center for Research Data

## Data Availability

The data sets generated and analyzed during this study are not publicly available due to the requirements of the Norwegian Center for Research Data to delete the data as soon as the processing is over. However, an anonymized version of the data set is available from the corresponding author on reasonable request.
